# High-efficiency targeted integration of extrachromosomal arrays in *C. elegans* using PhiC31 integrase

**DOI:** 10.1101/2025.11.11.687718

**Published:** 2025-11-12

**Authors:** Matthew S. Rich, Ryan Pellow, Adam Hefel, Ofer Rog, Erik M. Jorgensen

**Affiliations:** 1 Department of Biology, University of Utah; 2 HHMI

## Abstract

Extrachromosomal arrays are unique chromosome-like structures created from DNA injected into the *C. elegans* germline. Arrays are easy to create and allow for high expression of multiple transgenes. They are, however, unstable unless integrated into a chromosome. Current methods for integration, such as X-rays and CRISPR, damage DNA and are low-efficiency. Here, we demonstrate that the viral integrase PhiC31, which mediates a non-mutagenic recombination between short *attB* and *attP* sequences, can be used for extremely efficient and targeted integration of arrays.

In this method, a transgene, a selectable marker, and *attP* sites are injected into the gonad of a strain that (1) has an *attB* site in its genome, and (2) expresses PhiC31 in its germline. F1 extrachromosomal arrays are cloned, grown for multiple generations with selection, and then screened for homozygous array integrations. The procedure is simple, requires less time than screening for extrachromosomal arrays, and arrays can be screened for transgene function after stable integration. Arrays that transmit are integrated by PhiC31 with 50-95% efficiency, allowing for the isolation of many unique integrants from a single injection. Arrays can also be integrated at fluorescent landing pads and arbitrary sites in the genome. Using nanopore sequencing, we show that three new integrated arrays are between 1.6 and 18 megabases in length, assemble with large repeats, and can contain hundreds of copies of injected transgenes. We have built a collection of strains and plasmids to enable array integration at multiple sites in the genome using various selections. PhiC1-mediated Integration of Arrays of Transgenes (PhiAT) will allow *C. elegans* researchers to shift from using unstable extrachromosomal arrays to directly integrating arrays.

## Introduction

Extrachromosomal arrays are the workhorse of *C. elegans* transgenesis. These miniature chromosomes provide a simple and rapid way to overexpress transgenes; injecting a worm gonad with a DNA mix can yield dozens of unique mini chromosomes that segregate across cell divisions and generations (1). Injected DNA concatenates via homologous recombination and non-homologous end-joining (2, 3) into a megabase-scale circular mini-chromosome (4). Arrays form after fertilization, mature over the course of the first few cell divisions and are actively, but not perfectly, segregated by kinetochore proteins (5).

Because extrachromosomal arrays can be somatically lost and don’t follow Mendelian segregation, *C. elegans* geneticists have developed methods to integrate these giant molecules into the genome where they will be faithfully segregated. All current methods for integrating extrachromosomal arrays require DNA damage: double-stranded DNA breaks in both the genome and the array can be repaired by inserting the array into the genome. The most commonly used method to induce breaks is through irradiation (6, 7). In this method, extrachromosomal arrays are selected which have a transmission rate much less that 75%, then irradiated. Hundreds of F1 progeny are cloned and screened for 75% array transmission, indicating a heterozygous array in the genome (~1-5% of F1s). Those strains are then homozygosed to create a single integrated line. Irradiation breaks the genome very effectively, but randomly; arrays are integrated at random positions in the genome and must be mapped genetically. In addition, it is common for strains with arrays integrated by irradiation to have background mutations, ranging in size and severity from point mutations to chromosomal translocations. While many of these can be crossed out of the strain, because the endpoints of the integration site are often scrambled, it may be impossible to remove all background mutations.

The advent of CRISPR technologies enabled targeted integration that is less mutagenic than irradiation. Most methods create a double-stranded break in the genome (now at a single site of your choosing) and in the array (at potentially many targets encoded in the DNA that was injected) (8, 9). After introducing DNA breaks by CRISPR, various screening and selection strategies have been employed to isolate integrants. Targeting an endogenous gene provides a phenotypic selection; screening, for instance, for array(+) Dpys for arrays integrated at *dpy-3* or for array(+) benomyl-resistance for arrays integrated at *ben-1* (8). Fluorescent transgenes can be used as landing pads; integration knocks out the fluorescent protein, so integrants are isolated as array(+) and otherwise non-fluorescent (10). Alternatively, landing pads can be engineered with a split selectable marker for positive selection; for instance, unc-119(+) rescue occurs only when an array is integrated at the landing pad (9). Across the board, whether using X-rays or CRISPR, the efficiency of integration is low: generally between ~1-10% of animals after the integration process contain an integrated array in their genome. Because these methods rely on inducing breaks in both the genome and the array, they are mutagenic and the integrated array is not necessarily the same as its extrachromosomal progenitor. As such, there is still a need for an easy, high-efficiency, and non-mutagenic method for targeted integration of extrachromosomal arrays in *C. elegans*.

Phage have solved the problem of integrating their own DNA non-mutagenically into a host’s genome using serine integrases and short DNA sequences called attachment (att) sites (11). Virus genomes contain a single *attP* (“phage”) site; host genomes contain an *attB* (“bacterial”) site. A serine integrase encoded in the phage genome mediates a unidirectional recombination between the *attP* and *attB* sites, integrating the circular phage genome. This recombination converts the *attB* and *attP* sites to *attL* (“left”) and *attR* (“right”). The PhiC31 integrase (hereon referred to as “PhiC31”), is functional in the *C. elegans* germline. Recently, we and others have shown that PhiC31 can integrate single-copy *attP*-containing transgenes and protein tags at an *attB* site in the genome with high-efficiency (12-14). PhiC31 also has been shown to induce recombination between *attB* and *attP* sites located on homologous chromosomes (13). In this work, we show that PhiC31 can also integrate *attP*-containing extrachromosomal arrays at *attB* sites in the genome with very high efficiency. A single injection can yield a diverse collection of array sizes and compositions. We have named this method PhIAT (pronounced “fiat”) for **Ph**iC31-mediated **I**ntegration of **A**rrays of **T**ransgenes.

## Results

### Extrachromosomal arrays are integrated by PhiC31 at high efficiency.

**Figure 1.**
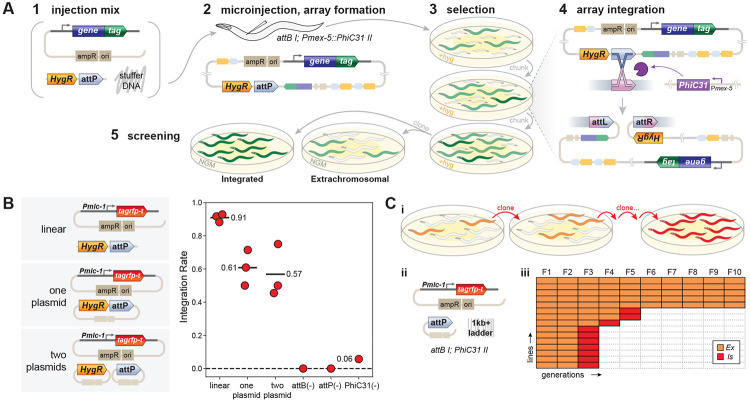
A method for targeted integration of extrachromosomal arrays using PhiC31. (A) Overview of PhIAT. (1) A DNA mixture is injected into the germline of a *C. elegans* strain that has an *attB* site in its genome and expresses PhiC31 integrase in its germline. (2) Array-positive F1s are cloned to hygromycin plates and (3) grown for multiple generations while selecting for array transmission. (4) During selection, the PhiC31 integrates the array into the genome. The generation after the array integrates, homozygous array(+) animals will be in the population and out-compete non-integrated animals. (E) After selection (in our hands, after 2-3 chunks), array(+) animals are screened for 100% transmission of the array. (B) Array integration efficiency. (left) Arrays were made by co-injecting a pan-muscle TagRFP-T transgene, a hygromycin selection, and *attP* site using either linear DNAs or plasmids. In plasmid-based integrations, the hygromycin selection and *attP* site were either contained on the same plasmid or split into different plasmids. (right) Integration rates were measured for linear and plasmid-based arrays as well as controls. Each point represents, for a single injection, the rate at which array(+) lines that survived selection yielded a homozygous integrant. The mean rate across experiments is show as a black bar. Controls used a linear injection mix. Linear: N=3 (n=12, 17, 14); one plasmid: N=3 (n=12, 23, 7); two plasmid: N=3 (n=11, 16, 20); *attB*(−): n=28; *attP*(−): 15; PhiC31(−): 35. (C) Array integration timing. (i) Outline of experiment. Array positive animals were cloned every generation, and their progeny screened for homozygous integrants. (ii) A linear injection mix was used in this experiment. Arrays were integrated into the *attB* site on chromosome I. (iii) A heatmap showing for each line whether the animals in that generation carried an extrachromosomal array (or non-homozygous integrant, orange) or an integrated array (red).

We tested whether PhiC31 could be used to integrate arrays into a single *attB* site in the *C. elegans* genome. DNA injected into a *C. elegans* gonad is assembled into large, circular extrachromosomal arrays that can contain many copies of each constituent DNA molecule. If arrays contain many *attP* sites, PhiC31 should mediate recombination between one of the *attP* sites and a single genomic *attB* site. Applying selection to maintain the array would cause animals carrying an integration event – even if it were rare and multiple generations after injection – to increase in frequency in the population, as most extrachromosomal arrays are lost between generations at significant rates. This method is summarized in Figure 1A.

To test the method, a strain was created that contained a single *attB* site on chromosome I (near *ttTi4348*, *oxSi1337[attB] I*) and a transgene expressing PhiC31 in the germline on chromosome II (*oxSi1347[Pmex-5::PhiC31(PATC)::SL2::mNeonGreen] II*). Into this strain a mixture of linear DNA fragments was injected: a PCR product of a pan-muscle TagRFP-T transgene (*Pmlc-1::TagRFP-T*), a hygromycin-resistance selectable marker to which we appended an *attP* site by PCR (HygR::*attP*), and stuffer DNA. Independent F1 array animals were singled and lines propagated at 25°C on hygromycin plates for about 10 generations (two rounds of starvation and chunking). After selection 8 animals per line were singled to permissive plates and screened for homozygous segregation of the RFP marker. Of the 12 lines that survived selection, 11 produced homozygous inserts (92% efficiency). 10 of these integrants were mapped and all mapped to chromosome I with recombination rates consistent with being inserted at the *attB* site (7.4cM from *dpy-5*; *oxSi1337* is 5.3cM from *dpy-5*). The mean integration rates of for arrays made from linear DNA was 91% from three independent experiments (Figure 1B).

Arrays can also be assembled from injection of circular plasmid DNA. Arrays generated from circular DNA were created in two ways: with the *attP* site and HygR on the same plasmid or on different plasmids. Both mixes used the pan-muscle TagRFP-T as a visible marker. These mixes were injected into the *attB PhiC31* strain, array-positive F1s singled to generate lines, and selection and screening performed as described above. Across three replicates of each experiment, arrays with a combined HygR::*attP* plasmid integrated in 61% of lines, and arrays with separate HygR and *attP* plasmids integrated in 57% of lines (Figure 1B).

We confirmed that integration was dependent on PhiC31. Integration was tested in a strain containing only a genomic *attB* site (‘no-PhiC31’), a strain only expressing PhiC31 (‘no-*attB’*), and using an array that lacked *attP* sites (‘no-*attP’*). Neither the ‘no-*attB’* nor the ‘no-*attP’* yielded integrations (out of 28 and 15 lines tested, respectively). Two arrays in the ‘no-PhiC31’ control experiment (out of 35 lines tested) yielded homozygous array insertions that did not map to the *attB* site on chromosome I (Figure 1B).

When does integration occur? Using the above selection, integration could occur at any point before screening at about the 10th generation after injection. To test the timing of integration we singled array(+) animals every generation and monitored integration by screening for homozygous arrays. The generation a homozygous array is picked is a conservative estimate for time of integration since that must have occurred in the germline of an animal at least two generations prior. Integration was tracked from 15 unique array lines over the course of 12 generations. (Figure 1C) Homozygous arrays were isolated from seven of the lines in the F3 generation, implying that these arrays integrated within the germlines of the F1. Three more lines integrated by the F5 generation. No integrations were isolated from the remaining five lines through the F10 generation.

### Strains and DNA reagents for targeted array insertion

**Figure 2.**
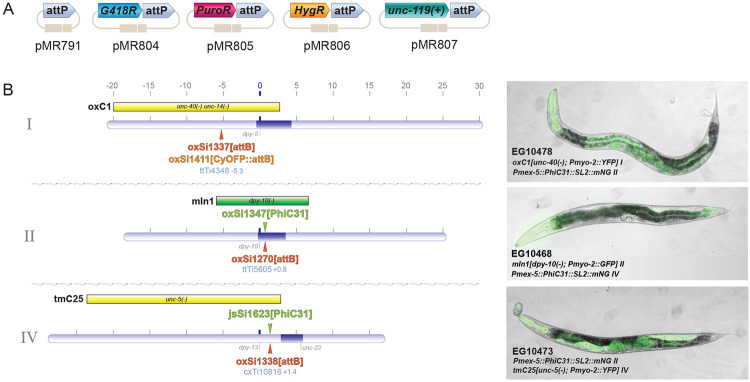
A collection of plasmids and strains for PhiC31-mediated array integration. (A) attP selection plasmids. pMR791 does not contain a selection but can be used in combination with any non-*attP* selection (e.g., *pha-1*, *lin-15*, *etc*). (B) Genetic map showing location positions of *attB* sites (orange), germline-expressed PhiC31 transgenes (green), and fluorescently-marked balancers with respect to chromosomes I, II, and IV. To the right of each chromosome is a representative image of the PhiC31(+) balancer(+) strain for each *attB* site. Fluorescence from the balancer is visible in the pharynx of each animal. In EG10478 (top) and EG10473 (bottom), mNeonGreen fluorescence marking the PhiC31 transgene (*oxSi1347 II*) is bright. In EG10438, the PhiC31 transgene (*jsSi1623 IV*) is dim and difficult to see along with bright fluorescence from the *mIn1* balancer.

To facilitate use of the method in the *C. elegans* community, we built various *attP* plasmids, as well as a set of *attB*- and PhiC31-containing strains. *attP* plasmids lacking a selection can be used in combination with any selection plasmid. We also created *attP* plasmids containing well-established drug resistance markers for hygromycin, neomycin/G418, and puromycin, as well as *unc-119(+)* rescue (Figure 2A). These plasmids were all tested and could successfully select for array integration. *attB* sites were created at safe-harbor loci on chromosomes I, II, and IV. In these strains, the *attB* sites were not marked. For PhiC31 transgenes, we tested our transgene on chromosome II (*oxSi1347*), and a transgene generated by Michael Nonet on chromosome IV (*jsSi1623 [Pmex-5::PhiC31::SL2::mNeonGreen] IV).* In our hands, both these transgenes are capable of high-efficiency integration (data not shown).

Although theoretically PhiC31 only acts on paired attB and attP sites, at a low probability PhiC31 binding to a lone attB site can lead to cuts and mutation of the site. Typically we inject directly into an *attB* PhiC31 strain. After propagating our original strain (*attB I* PhiC31 *II*) for about six months, integration stopped working; sequencing demonstrated that the *attB* site had been mutated (Supplemental Figure 1), presumably by long-term exposure to PhiC31. Thus, for long-term storage the *attB* site and PhiC31 must remain separated. A PhiC31-expressing strain can be crossed to an *attB* strain and homozygosed to reconstitute an injection strain.

To facilitate crossing, both the *attB* and PhiC31 strains were altered. An array was added to the *attB* strains that generates a high incidence of males (Him). Specifically, the array carries a piRNAi transgene that inhibits the *him-8* gene (15), and a fluorescent marker and HygR transgene for array selection The homozygous *attB* males segregated from this strain are crossed to the PhiC31 strain. The PhiC31 strains carry a fluorescent marker that balances the *attB* site in the F1 (Figure 2B). In the F2, *attB* homozygotes will lack the balancer, and PhiC31 can be homozygosed by following mNeonGreen germlines.

Do strains need to be homozygous for PhiC31 and *attB* to yield integrations? We tested whether heterozygous F1s generated in the cross above could be injected and yield integrations. Heterozygotes (*attB / mIn1 II; jsIs623[PhiC31, mNG] / + IV*) were injected with pan-muscle TagRFP-T and HygR::*attP* and assayed for array integration. Two injected animals were placed on each plate and hygromycin selection was applied for two chunks of growth. 5 out of 7 lines that survived selection yielded homozygous insertions. Thus, injecting F1 heterozygotes guarantees an undamaged *attB* target site with no loss in efficiency.

### Integrated arrays are diverse

**Figure 3.**
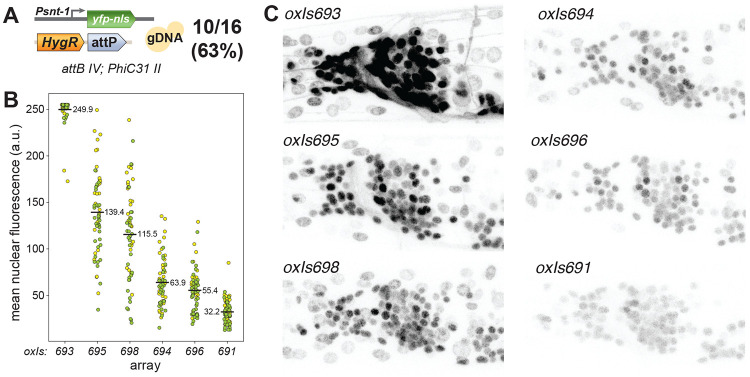
PhiC31-integrated arrays are diverse. (A) Injection mix for YFP arrays. PCR products for Psnt-1::YFP::histone and HygR::attP, along with yeast genomic DNA for stuffer, were injected into an PhiC31 II; attB IV strain. 10/16 lines that survived hygromycin selection generated integrants in this experiment. (B) Animals with integrated arrays were imaged by confocal microscopy to measure the YFP brightness of their neuronal nuclei. The brightness of nuclei in the anterior ganglion were quantified. Each point denotes a single nucleus. Colors (yellow or green) denote measurement from two individuals. The mean fluorescence across all nuclei is annotated. (C) Representative images of integrants, YFP is largely restricted to nuclei; however fluorescence from the brightest arrays (*oxIs693* and *oxIs695*) was observed leaking into neurites. The *oxIs693*, *oxIs695*, and *oxIs691* arrays were sequenced and assembled.

Independent arrays usually have a range of expression levels from transgenes, and individual arrays must be carefully selected for optimal expression. We tested whether integrants from a single injection also generated a range of expression levels of a fluorescent transgene. 10 arrays containing pan-neuronal YFP (*Psnt-1::YFP::histone*) at the *attB* site on chromosome IV from were isolated 16 array-bearing lines (63%, Figure 3A). The expression levels of YFP from these arrays ranged eight-fold from the brightest to dimmest arrays (Figure 3B). While the majority of YFP fluorescence was limited to neuronal nuclei, the brightest arrays exhibited fluorescence leaking into the commissures (Figure 3C), potentially due to overwhelming nuclear import. YFP was also weakly expressed in non-neuronal nuclei in most arrays, perhaps due to leak from the YFP transgene, which contained a 3’ UTR from muscle-expressed gene *unc-54*.

### Long-read assemblies of integrated arrays

The structure of extrachromosomal and integrated arrays has been characterized largely at the medium-scale by Southern blots (1, 2) and imaging (4). Arrays are thought to be highly repetitive, which complicates sequencing and assembly using short-read sequencing methods. Recently, groups have sequenced and attempted to assemble arrays using long-read nanopore sequencing (9, 16, 17), though none of these efforts were able to yield fully assembled array sequences. To increase fragment length, and therefore assembly quality, we optimized an extraction protocol for high molecular weight DNA (see Methods) and performed nanopore DNA sequencing.

**Figure 4.**
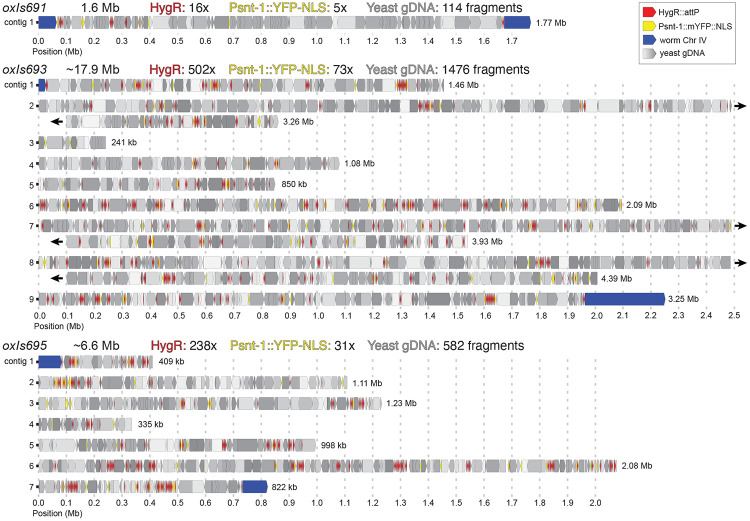
Assembled integrated arrays. Annotated contigs for *oxIs691, oxIs693, and oxIs695*. The estimated total length of each array, as well as number of constituent fragments, is written above each diagram. A single contig is drawn per line. In *oxIs693* contigs 2, 7, and 8, were too long to plot easily and were wrapped to new lines; these cases are denoted with black arrowheads. The length of each contig is noted at the end of each line. An additional megabase of *C. elegans* sequence has been truncated from contig 9 in *oxIs693*. Annotations are drawn as boxes with arrows pointing in the direction of transcription (for transgenes) or based on the reference (for yeast or worm genome fragments) and are colored based on their identity (HygR: red; YFP: yellow, *C. elegans* chromosome IV: blue; yeast genomic DNA: gray, shaded based on chromosome).

The RFP arrays used DNA ladder as stuffer DNA, which proved too short and repetitive to be assembled (*oxIs675*, not shown). Thereafter, we used yeast genomic DNA as a stuffer for the pan-neuronal YFP arrays. This DNA provided large fragments with high-sequence diversity to the array. Three integrated arrays (*oxIs693, oxIs695, and oxIs691*) were chosen to span the range of expression levels from the set. High molecular weight DNA was extracted and sequenced using the Oxford Nanopore MinION platform (sequencing statistics can be found in Table S4). Annotated gene models of each array are found in Figure 4. The length of these arrays vary from 2 Mb to 18 Mb and sequences content accounts for differences in transgene expression:

#### oxIs691.

This is the dimmest array of the set. *oxIs691* is ~1.6Mb long and contains 3 full-length YFP fragments, 16 full-length HygR fragments, and 111 yeast fragments. We obtained ~279K reads longer than 10kb and fully assembled the array in the expected location on chromosome IV flanked by *attL* and *attR* sites. Yeast gDNA fragments ranged from 76-65673bp in length, with 91.5% being shorter than 1000bp. 2.3% of the array was made of HygR fragments, 1.6% was made of YFP fragments, and 96.1% was the yeast genome. Yeast fragments in the array covered 9% of the total yeast genome.

#### oxIs695.

This is the second-brightest array. oxIs695 is ~6.8Mb long and contains 31 full-length YFP fragments, 238 full-length HygR fragments and 558 yeast fragments. We obtained 185K reads longer than 10kb and were able to assemble this array into seven contigs ranging from 335kb to 2.1Mb (N50=998kb). The array integrated in the correct location on chromosome IV flanked by *attL* and *attR* sites. Yeast gDNA fragments ranged from 45-74829bp in length; 86% were shorter 1000bp. 8.1% of the array was made of HygR fragments, 3% was made of YFP fragments, and 88.9% was the yeast genome. Yeast fragments in the array covered 31% of the total yeast genome.

#### oxIs693.

This is the brightest array. oxIs693 is ~17.9Mb long and contains 73 full-length YFP fragments, 502 full-length HygR fragments and 1446 yeast fragments. We were able to assemble this array into nine contigs ranging from 240kb to 4.4Mb (N50=2.3Mb). The array integrated in the correct location on chromosome IV flanked by *attL* and *attR* sites. Yeast gDNA fragments ranged from 43-85495bp in length; 83% were shorter 1000bp. 5.8% of the array was made of HygR fragments, 2.2% was made of YFP fragments, and 92% was the yeast genome. Yeast fragments in the array covered 55% of the total yeast genome.

Combined, the three arrays covered 73% of the yeast genome (8.2 Mb of the 11.2 Mb yeast genome). Fragments came from all 16 yeast chromosomes and the mitochondrial DNA. 95% of the bases of the yeast genome were present fewer than five times in the array, as would be expected from randomly incorporating sheared genomic DNA into the array (Supplementary Figure 2A). Surprisingly, an identical fragment from ChrXII (XII:451,417-468,929) was present multiple times in all three arrays. This fragment was mapped 29 times in *oxIs691,* 51 times in *oxIs695*, and 9 times in *oxIs693*. This portion of the yeast genome is a tandem duplication flanked by autonomously-replicating sequences (Supplementary Figure 2B-C), suggesting they may be present as multicopy circular DNA or integrated repeats in the original yeast strain.

We were unable to fully assemble the two largest arrays *oxIs693* and *oxIs695* due to the presence of large duplications within the array. Each array has 21 regions longer than 50kb that are duplicated in multiple contigs (Supplemental Figure 3). Repeats are composed of multiple DNA fragments, implying that they were formed initially, then duplicated during array assembly.

Arrays formed from circular DNAs consist of tandem arrays of injected DNAs, while arrays made from linear DNAs assort randomly by end-joining (9, 17, 18). At first glance, the assembly of our arrays (made from linears) is consistent with random assortment, though we noticed that the HygR fragments appeared to be clustered. We tested whether they were clustered using a Monte Carlo simulation in which the order of the fragments in the arrays were randomly assorted and then scored for whether neighbors were the same fragment type (yeast, HygR, or YFP). The HygR transgenes were significantly clustered (p<1e-5 in both arrays), while YFP transgenes in the array were randomly spaced (p=0.16 in *oxIs695* and p=0.42 in *oxIs693*).

### One-shot attB and array integration

**Figure 5.**
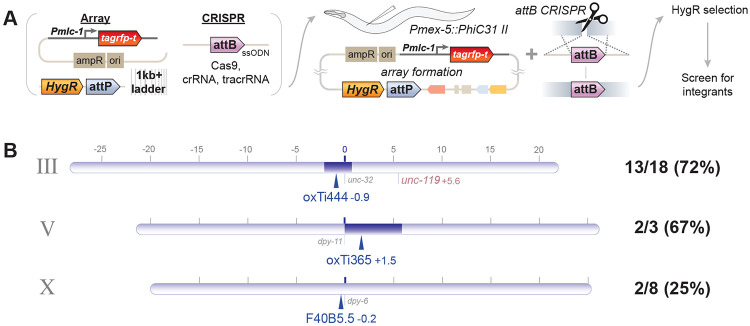
attB knock-in and array integration from a single injection. (A) Overview of method. A PhiC31(+) strain is injected with a single mix containing DNA reagents for an array and CRISPR reagents for knocking an attB site into the genome with and ssODN and Cas9 ribonucleoprotein complex. Array(+) F1 animals, which have also likely received CRISPR reagents that can insert an attB site in the genome, are cloned, selected, and screened for integrants. (B) Genetic map for three sites in the genome where we tried this method: near the oxTi444 locus on chromosome II, near the oxTi365 locus on chromosome V, and near F40B5.5 on chromosome X. The frequency at which we isolated integrants at each site is shown to the right of the chromosome.

The strains built for this manuscript contain *attB* sites near common landing pads on chromosomes I, II, and IV. *attB* sites are short and simple to edit into the genome by injecting Cas9 ribonucleoprotein complex and a single-stranded DNA repair donor (19). We tested whether we could integrate an array at an arbitrary site in the genome through two sequential steps from a single injection: (1) edit an *attB* site into the genome using CRISPR reagents, then (2) integrate an array formed from co-injected DNA at that site (Figure 5A).

A PhiC31-expressing strain was injected with the CRISPR reagents to insert an *attB* site on chromosome III near the location of *oxTi444* universal mosSCI insertion site. The injection mix also contained PCR products for pan-muscle TagRFP-T and HygR::*attP*. F1 array(+) animals were cloned and selected. Of the 18 lines that survived selection, 13 yielded a homozygous array integration (72%). Genetic mapping of these insertions placed them on chromosome III consistent with being inserted at the new *attB* site (0.9cM away from *unc-32*, *oxTi444* is 0.9cM from *unc-32*). The same experiment was repeated targeting sites on chromosomes V (near the *oxTi365* universal MosSCI site) and X (within the *F40B5.5* ncRNA cluster), yielding 2 integrants from 3 lines with an insertion (67%) for chromosome V and 2 integrants from 8 lines (25%) for chromosome X (Figure 5B). These experiments show that arrays can be inserted at new *attB* sites using a single injection.

### Integration at a fluorescent landing pad

We have focused on integrating at simple *attB* sites that are otherwise unmarked, primarily because these are very easy to knock into the genome. Because *attB*s are dark, screening for integrations can be done only by visually testing for homozygous transmission. In FLInt (10), another method for targeted integration, arrays are knocked into a fluorescent transgene. Integration of the array at the landing pad knocks out the landing pad’s fluorescence. This provides both a positive screen (presence of the array) and negative screen (loss of landing pad fluorescence).

**Figure 6.**
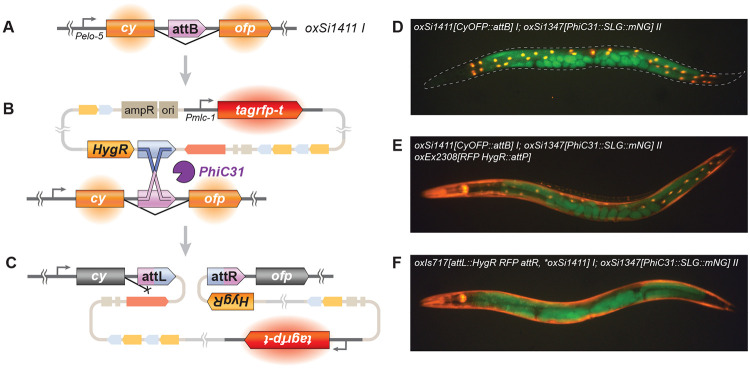
Integration into a fluorescent landing pad. (A-C) Schematic of integration into a landing pad. (A) A landing pad containing an intestinally-expressed nuclear-localized CyOFP is integrated as a single copy in the genome (in this case, on chromosome I). An attB site has been inserted in an early intron in the transgene. (B) An extrachromosomal array (in this case, containing muscle-expressed TagRFP-T attP sites and hygromycin selection) is injected into the landing pad strain. During selection, PhiC31 mediates recombination between attP in the array and attB in the genome. (C) When the array is integrated, it is recombined into the CyOFP intron, knocking out the fluorescence. Fluorescence from the array is maintained. (D-F) Representative images of animals at each stage in the integration process. In (D), CyOFP(+) intestinal nuclei can be seen. In (E), the strain carries an extrachromosomal array before integration; both CyOFP(+) intestinal nuclei and TagRFP-T fluorescence from the array can be seen. In (F), the array from (E) has integrated into the landing pad and is homozygous; no CyOFP fluorescence is present in the intestine. Germline mNeonGreen fluorescence from the PhiC31 transgene is present in all images.

We implemented this strategy with our method. A new landing pad was created, containing a nuclear--localized CyOFP transgene expressed in the intestine (Figure 6). An *attB* site was inserted within an intron in the *cyofp* gene. A single copy of this landing pad was integrated on chromosome I and crossed to a strain expressing PhiC31. This strain was injected with an array mixture containing a linear pan-muscle TagRFP-T transgene and a HygR::*attP* PCR product, and array(+) lines were selected on hygromycin as before. In addition to screening for inserts post-selection (our standard screening method), lines were examined after each chunk for array(+) CyOFP(−) animals. Out of 25 lines, integrations in 11 lines were isolated using the positive-negative visual screen (44% integration rate).

One additional line integrated its array, but did not lose CyOFP fluorescence. Another line lost CyOFP fluorescence but did not integrate its array. These experiments show that arrays can be integrated into a fluorescent transgene at similar rates to integration at a “naked” *attB*.

## Discussion

For extrachromosomal arrays to be maintained with stable expression and segregation, they must be integrated into the genome. PhIAT is a simple method for high-efficiency targeted integration of extrachromosomal arrays in *C. elegans*. Arrays that contain PhiC31 *attP* sites are integrated with 50-100% efficiency into a genomic *attB* site. This method is simple and can yield large numbers of diverse integrated arrays at specified loci with little researcher effort.

Currently the best methods for targeted array integration use CRISPR to cut the genome; mosTI (9) and FLInt (10) generate integrations in 25% and 3% of array-bearing progeny, respectively. PhiC31 integrates extrachromosomal arrays with very high efficiency, greater than 90% of the array bearing lines for linear arrays. Ironically, the improved efficiency may be due to the sluggish inefficiency of PhiC31. In both FLInt and mosTI, array formation is occurring at the same time as Cas9 cleavage of the integration site. If an array isn’t formed and inserted into the cut site before the double-strand break is repaired and mutated, that line is a dead-end. Injected Cas9 ribonucleoprotein complex is short-lived and lost after the P0 generation. Plasmid reagents delivered by mosTI injection can persist through generations, but the array must have incorporated the Cas9 transgene. By contrast, in our method, the extrachromosomal arrays form after injection, and then expression of PhiC31 integrates the array at some point in subsequent generations.

Should all arrays be integrated by PhIAT? Maybe not. In our hands, PhIAT works very well for integrating arrays that do not affect the physiology of the worm (for instance, fluorescent reporters and tagged proteins). PhIAT may struggle with arrays that are toxic (whether purposefully, when overexpressing dominant negative transgenes, or incidentally, as for arrays that squelch because they contain too much *Pmyo-2*). The method uses selection to increase an integrated array’s frequency in the population; selection against array toxicity fights against selection for array transmission. We have not intentionally tested whether PhIAT integrates toxic arrays with high efficiency, but have isolated some arrays that cause the worm to be sick. Integrating toxic arrays soon after injection is an effective strategy (21), so using other methods in which integration is screened early will likely be more useful than PhIAT for targeted integration.

A benefit of PhIAT is the number of integrants one can isolate. Here, we isolated as many as 24 unique integrated arrays from a single injection session, and we noted that integrants had diverse ratios of incorporated DNA. Array diversity is important for obtaining the correct stoichiometry of transgenes. For example, the composition of the NeuroPAL array, used for identifying neurons by color and position, needed to be tuned to optimize expression of four fluorescent proteins across 41 transgenes (20). With PhIAT, it seems feasible that with only moderate effort at the injection microscope one could create even hundreds of stably integrated arrays. Isolating the one perfectly stoichiometric array is only a matter of screening for function.

We sequenced and assembled three integrated arrays using long-read sequencing. To our knowledge, the assembly of *oxIs691* is the first end-to-end assembly of a large repetitive array (a ~35kb biolistic insertion has also been assembled end-to-end using nanopore sequencing (16)). We were able to achieve this by improving array assembly in two ways: First, we optimized a protocol for extraction of high molecular weight DNA from *C. elegans*. Good assemblies require sequencing very long DNA molecules in order to scaffold repetitive contigs. Over 50% of our nanopore reads were longer than 15kb, and some were over 150kb in length, which gave us sufficient coverage to use only reads longer than 50kb in our assemblies. Second, after computational assembly, we further refined contigs using hand-assembly using polymorphisms and incomplete DNA fragments (for example, *Psnt-1::YFP* transgenes that did not include their histone sequences) as landmarks. Our assemblies supplement the small but growing set of sequenced arrays in *C. elegans* (9, 17), and provide insight into possible mechanisms through which the array forms.

Finally, we note that the size of the arrays we and others have integrated are on the scale of whole microbial genomes. Our own arrays incorporate 72% of the entire yeast genome. *C. elegans*, therefore, could be used as a crucible for studying the evolution and function of genomes of other organisms, transgressing the boundaries between species.

## Materials and Methods

### Reagents

All chemicals were purchased from Sigma-Aldrich (St. Louis). Hygromycin, G418, and puromycin were purchased from GoldBio (St. Louis). All enzymes were purchased from New England Biolabs (Beverly, MA). All synthetic DNAs were purchased from Integrated DNA Technologies.

### C. elegans strains

*C. elegans* strains were cultured using standard methods (22) on nematode growth media (NGM) feeding on OP50 or HB101 bacteria. Animals were maintained at 15°C, 20°C, and 25°C. A list of worm strains created in this study can be found in Table S1. Drug plates were made by top-plating 500ul of drug (hygromycin: 4mg/ul, G418: 25mg/ul, puromycin: 10mg/ml + 0.1% Triton X-100) onto 6cm NGM+HB101 plates.

### Molecular biology and cloning

A list of plasmids used in this study can be found in Table S2. A list of primers and RNAs used in this study can be found in Table S3. Novel plasmids were created using standard molecular biology techniques or ordered from Twist Bioscience. Annotated sequences for all plasmids can be found in Supplementary File 2.

### General injection procedures

All injections were conducted into young adult (<48 hours) hermaphrodites raised at 15°C on HB101 bacteria. All plasmids were purified using the Qiagen miniprep kit (Qiagen). Linear DNAs were prepared either by PCR using Q5 polymerase (NEB) or plasmid digestion followed by gel extraction using the Qiagen Minelute gel extraction kit (Qiagen).

### Integration method

An injection mix containing transgene cargo, an attP-containing fragment/plasmid, a selection fragment/plasmid, and stuffer DNA was injected into young adult hermaphrodites that have an attB in their genome and express PhiC31 in their germline. Stuffer DNA was either a 1kb+ DNA ladder (Invitrogen #10787018) or yeast genomic DNA (Sigma-Aldrich #69240-3). Injected animals were pooled 2-3 per NGM plate. After three days, array(+) F1s were cloned to selective plates (or NGM plates in the case of *unc-119* rescue) and grown at 25°C. When plates were close to starvation, a 1cm x 1cm chunk was moved to a new selective plate. Plates can be chunked when close to starvation as many times as are necessary. In this manuscript, after two chunk’s-worth of growth, strains were chunked to non-selective plates to grow animals for screening. The next day, 8 array(+) L2-L4 animals per line were cloned to non-selective plates. Three days later these plates were screened for homozygous array(+) progeny. Results from all integration experiments can be found in File S1.

### Integration without selection

EG10476 and EG10469 were crossed to reconstitute an *oxSi1337[attB] I; oxSi1347[PhiC31] II* strain. These animals were injected with pSEM233 (at 1ng/ul, linearized by NdeI digest), pMR791 (at 10ng/ul, linearized by NotI digest) and 1kb+ DNA ladder (at 75ng/ul). F1 array(+) progeny were cloned to NGM plates. Each generation (~ every three days), 4 animals from each line were cloned to new NGM plates to create a set of four plates for each line. Plates from each line were screened for 100% array(+) progeny at each generation.

### attB CRISPR and integration

EG10408 (*oxSi1347[PhiC31] II*) was injected with Cas9 (325ng/ul final concentration, UC Berkeley QB3 Macrolab), tracrRNA (100 pmol, IDT), an sgRNA for an insertion site (100 pmol, IDT), an ssODN for repairing with an attB site (100 pmol, IDT), and 100ng/ul of array cargo (see Table S3 for crRNA and ssODN sequences). The selection and screening strategy was the same as for injection into a PhiC31, attB strain: array(+) F1s were cloned to hygromycin plates and grown at 25C for cycles of starvation and chunking. 8 animals for each line were then cloned to NGM plates and screened for homozygous integrated arrays.

### Creation of oxC1.

The recombination suppressor balancer *tmC20* balances the attB site on chromosome I. This balancer has an integrated array linked to *unc-14*, but has no phenotype. In order to add a recessive mutation with a visible phenotype to *tmC20*, we created a premature stop in *unc-40*. We injected EG10476 (*tmC20 I; oxSi1347[PhiC31] II*) with CRISPR reagents and fluorescent co-injection markers targeting residue 1138 in *unc-40* and screened the progeny of array(+) F1s for *unc-40(−)* F2s. These were cloned and outcrossed to N2 to (1) isolate them from the PhiC31 transgene in the injection strain background, and (2) confirm that the *unc-40* mutation was linked to the Pmyo-2::YFP fluorescent marker present in the balancer.

### Fluorescent imaging.

Confocal images were taken using a Zeiss LSM880 using a GaAsP detector. Epifluorescent images were taken on a Zeiss Axioskop compound fluorescence microscope equipped with a CoolLED pe-300lite white light LED and an Amscope MF2003-BI color CMOS camera. Worms were anesthetized using 50mM sodium azide before mounting on a 3% agarose pad for imaging. For images where direct comparisons were required, the same imaging settings were used for all samples.

### DNA extraction for whole-genome shotgun sequencing

*C. elegans* lines were grown on 10cm NGM plates seeded with HB101 bacteria. Before starvation, worms were gently washed off plates with M9 and pelleted and washed 2x with M9 to remove bacteria. Genomic DNA was extracted using a Qiagen DNAeasy Blood and Tissue kit (Qiagen). Whole genome sequencing was performed by the Huntsman Cancer Center Genomics Core. Libraries were prepped using the New England Biolabs NEBNext Ultra II FS DNA Library Prep kit (cat#E7805L) and sequenced on an Illumina NovaSeq X using a paired-end 2×151cycle protocol.

### Identifying array insertions from short read sequencing

FASTQ files were processed using a custom pipeline. Briefly, reads were mapped using bwa-mem (23) to a reference comprised of the *C. elegans* genome (WS276) and plasmid sequences contained in the injection mix of the sequenced strain (usually pSEM233 and pCFJ782). Mapped reads were filtered using samtools (24) for fragments that were split between a *C. elegans* chromosome and one of the plasmids. These reads were then hand-examined using IGV (25) to confirm the insertion site.

### DNA extraction for nanopore sequencing

*C. elegans* transgenic were grown to starvation on a 15cm 2XYT media plate that was seeded with NA22. Starved worms were rinsed off the plate into a 15mL conical with wash buffer (100mM Tris-HCl pH 8.0, 100mM NaCl, 20mM EDTA). To further remove any residual bacteria, worms were pelleted and washed three times with wash buffer, with the middle wash including a 30-minute incubation at room temperature to aid the removal of any gut bacteria. After pelleting, 50μL of worms were resuspended in 485μL of wash buffer and frozen at −80°C (16). The worms were subsequently thawed and Proteinase K and SDS were then added to final concentrations of 4 mg/mL and 0.2%, respectively. Samples were then incubated for 2 hours at 60°C. Following this initial digestion, 500μL of lysis buffer (100mM Tris-HCl pH 8.0, 100mM NaCl, 20mM EDTA, 4mg/mL Proteinase K and 0.2% SDS) was added and the sample was incubated overnight. RNAse was then added, and the sample was incubated at 30°C for 30 minutes. DNA was then extracted by performing a phenol-chloroform extraction and ethanol precipitation (26-28). DNA quality and quantity was checked using a NanoDrop spectrophotometer and a Thermo Fisher Scientific Qubit 4 Fluorometer.

Oxford Nanopore Technologies (ONT) libraries were prepared using ONT SQK-LSK114 according to refs (16, 27). All samples were sequenced to over 50x coverage (>5Gb) using the ONT MinION sequencing device based on manufacturer’s instructions and previously published protocols (16, 27). All basecalling was performed using the ONT MinKNOW software and the super-accurate basecalling model, with only passed reads and reads over 1kb being kept for downstream analysis. Sequencing quality was analyzed using NanoPlot (v.1.41.3) (29).

### Assembly of array reads

Integrated arrays were assembled began by performing *de novo* genome assembly using Flye (v.2.9.1-b1780) (30) with the following parameters; --genome-size 120m --threads 56 --iterations 5 --min-overlap 10000 --asm-coverage 40. Locations of the integrated arrays were confirmed by mapping the Flye contigs to *C. elegans* reference genome (WBcel235) (31). The quality and completeness of each assembly was manually inspected using IGV (v.2.16.1) (25). When necessary, integrated array assemblies were corrected or extended using a combination of Winnowmap2 (v.2.03) (32) followed by Flye for polishing. More specifically, this meant mapping all reads to the integrated array contigs and checking for any soft-clipped reads in IGV that could be used to extend contigs. If the integrated array contigs could be extended, the sequences from the soft-clipped reads were then manually concatenated to the end of the contig and validated using Winnowmap2 and IGV. If correct, the new additions were polished using Flye. A similar method for elongating contigs was employed by (33). The sequences of the pCFJ782, pMR209, the *C. elegans* reference genome (WBcel235) (31), and the *S. cerevisiae* reference genome (R64-sacCer3) (34) were then mapped to the integrated array contigs using minimap2 (35).

### Analysis of array assemblies.

All analyses were performed using custom Python scripts (available upon request). Plots were created using matplotlib (36) and seaborn (37). To identify repeats within assemblies, contigs were aligned to each other using minimap2 using default parameters. The resulting hits were filtered for those longer than 50kb and with a mapping quality score greater than 50. Circos plots were drawn using pyCirclize (https://github.com/moshi4/pyCirclize).

## Supplementary Material

Supplement 1

Supplement 2

Supplement 3

4
