# Supplementary material for: High-efficiency targeted integration of extrachromosomal arrays in C. elegans using PhiC31 integrase
Source: bioRxiv. 2025 Nov 12:2025.11.11.687718. Preprint. [Version 1] doi: 10.1101/2025.11.11.687718 (PMC12642426; doi:10.1101/2025.11.11.687718)
Supplement: 4 [file NIHPP2025.11.11.687718v1-supplement-4.pdf]

bioRxiv preprint doi: https://doi.org/10.1101/2025.11.11.687718; this version posted November 12, 2025. The copyright holder for this preprint (which was not certified by peer review) is the author/funder, who has granted bioRxiv a license to display the preprint in perpetuity. It is made available under aCC-BY 4.0 International license.

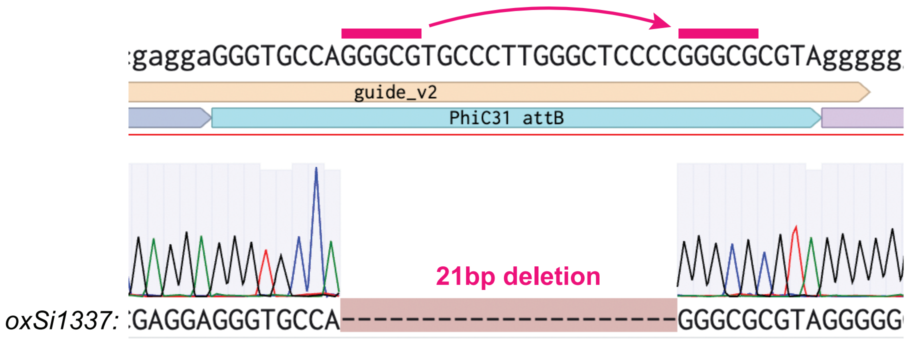

Supplemental Figure 1. Mutation to attB site after long-term propagation with PhiC31. A strain combining the *oxSi1337 I* attB site with *oxSi1347[PhiC31] II* was propagated for over six months, at which point the strain did not integrate arrays. We sequenced the attB site and found it contained a 21bp deletion (shown above as a screenshot from an alignment in Benchling). Above the reference sequence (top) is noted two GGGCG sequences contained within the attB site; it is possible that recombination between these sites is due to microhomology-mediated or theta-mediated end joining repair after a nick or double stranded break.

bioRxiv preprint doi: https://doi.org/10.1101/2025.11.11.687718; this version posted November 12, 2025. The copyright holder for this preprint (which was not certified by peer review) is the author/funder, who has granted bioRxiv a license to display the preprint in perpetuity. It is made available under aCC-BY 4.0 International license.

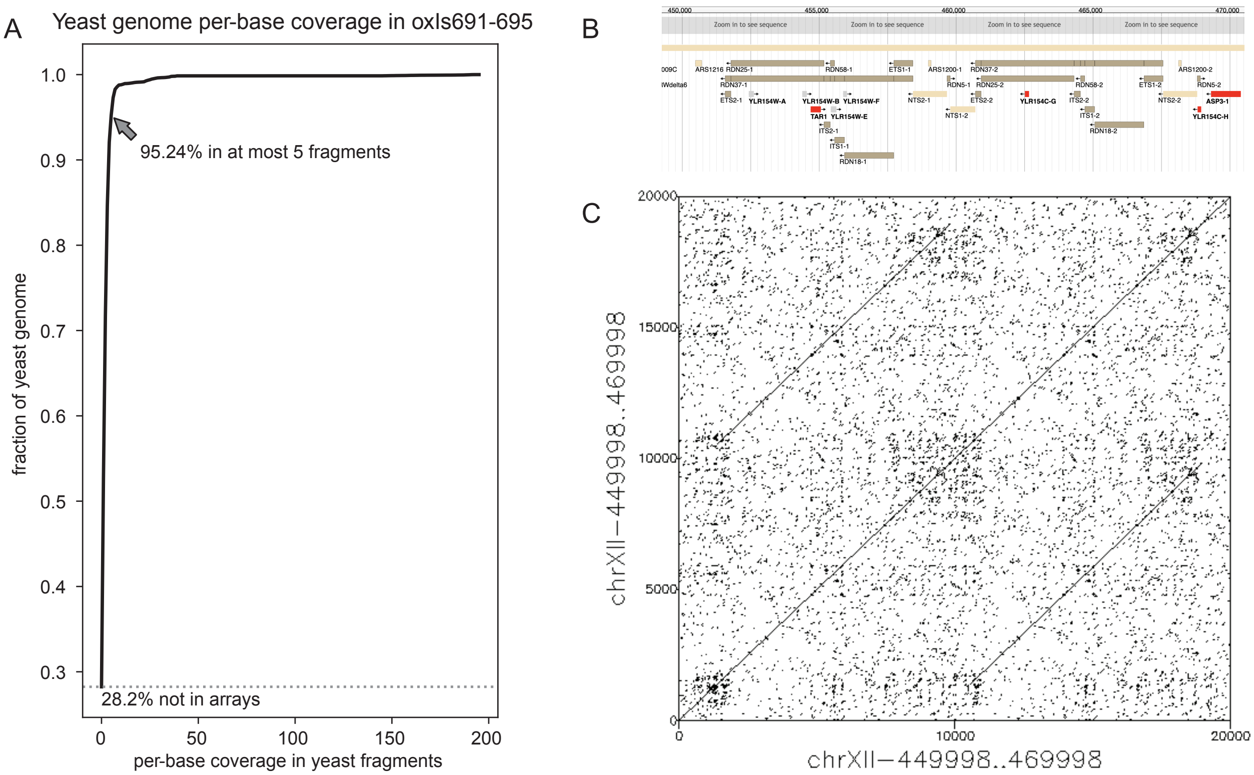

Supplemental Figure 2. Coverage of the yeast genome in integrated arrays. (A) Cumulative plot showing how often a base in the yeast genome is present is our arrays. 28.2% of the genome is not present in the arrays and over 95% of the yeast genome is represented in between 1 and 5 fragments across all three arrays. The long tail in the distribution up to bases found up to 195 times in the array, is caused by the repeated incorporation of a fragment from ChrXII. A browser shot of the region is shown in (B). (C) A dot plot of the region. The diagonal lines in the plot denote that it is a tandem duplication. Dot plot made by dotmatcher using the following parameters: windowsize=20, threshold=40.

bioRxiv preprint doi: https://doi.org/10.1101/2025.11.11.687718; this version posted November 12, 2025. The copyright holder for this preprint (which was not certified by peer review) is the author/funder, who has granted bioRxiv a license to display the preprint in perpetuity. It is made available under aCC-BY 4.0 International license.

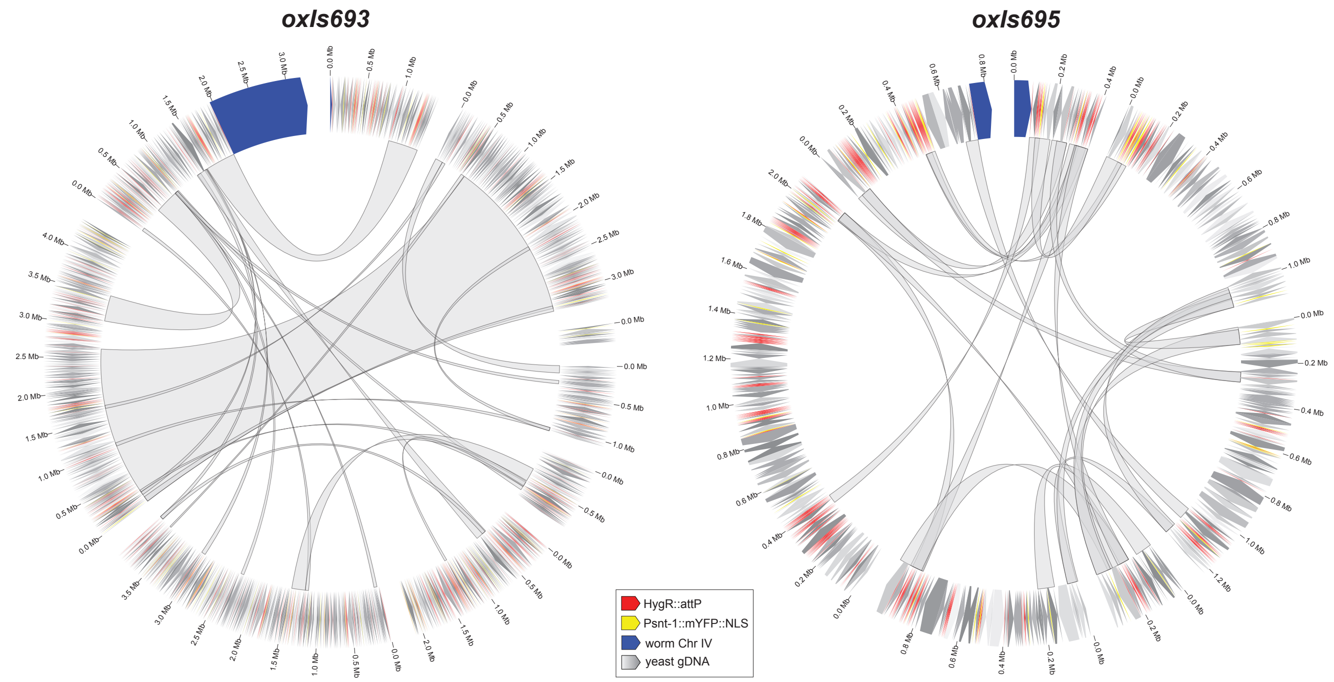

Supplemental Figure 3. Large repeats found in integrated arrays. Repeats that are found on multiple contigs are plotted as Circos plots for *oxIs693* (left) and *oxIs695* (right). Around the outside of the plots, the annotated array sequence is plotted as in Figure 4. Arcs inside the plot denote repeats longer than 50kb. Both arrays have 20 of these repeats. The large repeat in *oxIs693* is likely a missed merger between contigs during assembly.
